# The scent of cuteness—neural signatures of infant body odors

**DOI:** 10.1093/scan/nsae038

**Published:** 2024-06-08

**Authors:** Laura Schäfer, Carina Köppel, Denise Kreßner-Kiel, Sarah Schwerdtfeger, Marie Michael, Kerstin Weidner, Ilona Croy

**Affiliations:** Department of Psychotherapy and Psychosomatic Medicine, Faculty of Medicine and University Hospital Carl Gustav Carus, TUD Dresden University of Technology, Fetscherstraße 74, Dresden 01307, Germany; Department of Psychotherapy and Psychosomatic Medicine, Faculty of Medicine and University Hospital Carl Gustav Carus, TUD Dresden University of Technology, Fetscherstraße 74, Dresden 01307, Germany; Department of Psychiatry and Psychotherapy, Charité—Universitätsmedizin Berlin, Freie Universität Berlin and Humboldt-Universität zu Berlin, Berlin 12203, Germany; Department of Psychotherapy and Psychosomatic Medicine, Faculty of Medicine and University Hospital Carl Gustav Carus, TUD Dresden University of Technology, Fetscherstraße 74, Dresden 01307, Germany; Department of Psychotherapy and Psychosomatic Medicine, Faculty of Medicine and University Hospital Carl Gustav Carus, TUD Dresden University of Technology, Fetscherstraße 74, Dresden 01307, Germany; Max Planck School of Cognition, Leipzig 04103, Germany; Department of Comparative Cultural Psychology, Max Planck Institute for Evolutionary Anthroplogy, Leipzig 04103, Germany; Department of Psychotherapy and Psychosomatic Medicine, Faculty of Medicine and University Hospital Carl Gustav Carus, TUD Dresden University of Technology, Fetscherstraße 74, Dresden 01307, Germany; Department of Psychotherapy and Psychosomatic Medicine, Faculty of Medicine and University Hospital Carl Gustav Carus, TUD Dresden University of Technology, Fetscherstraße 74, Dresden 01307, Germany; Department of Clinical Psychology, Institute of Psychology, Friedrich-Schiller-Universität Jena, Jena 07737, Germany; German Center for Mental Health (DZPG), Site Jena-Magdeburg-Halle 07737, Germany

**Keywords:** chemosignals, mother–infant relationship, Kindchenschema, chemosensory communication, body odor

## Abstract

The smell of the own baby is a salient cue for human kin recognition and bonding. We hypothesized that infant body odors function like other cues of the Kindchenschema by recruiting neural circuits of pleasure and reward. In two functional magnetic resonance imaging studies, we presented infantile and post-pubertal body odors to nulliparae and mothers (*N* = 78). All body odors increased blood-oxygen-level-dependent (BOLD) response and functional connectivity in circuits related to olfactory perception, pleasure and reward. Neural activation strength in pleasure and reward areas positively correlated with perceptual ratings across all participants. Compared to body odor of post-pubertal children, infant body odors specifically enhanced BOLD signal and functional connectivity in reward and pleasure circuits, suggesting that infantile body odors prime the brain for prosocial interaction. This supports the idea that infant body odors are part of the Kindchenschema. The additional observation of functional connectivity being related to maternal and kin state speaks for experience-dependent priming.

## Introduction

Cuteness serves an infant’s innate mechanism for ensuring survival by evoking nurturance and affection from the caregiver (Kringelbach *et al*., 2016). The initial concept of cuteness originates in Konrad Lorenz’s ‘Kindchenschema’ and refers to infantile visual features (Lorenz, 1943; Tinbergen, 1963). It describes the prototypical appealing and adorable features of a baby’s face, such as big eyes, small nose and plump cheeks. These characteristics constitute the Kindchenschema, an ‘innate releasing mechanism’ (Lorenz, 1943) that instinctively triggers positive responses in the recipient (e.g. cuddling and comforting) to protect, nurture and care for the child. These responses directly benefit the infant by ensuring its survival and thereby also benefit the survival of the species (Gillam, 2011). The preference of and reaction to Kindchenschema cues are well investigated. Adults prefer baby as compared to adult faces (Brosch *et al*., 2007; Parsons *et al*., 2011), and infant cuteness affects the motivational and emotional state. Glocker *et al*. (2009), for instance, demonstrated that those baby faces that are rated as cuter also elicit higher motivation to care.

In principle, infant faces evoke similar responses of positive affect in parents and non-parents (Senese *et al*., 2013). However, parents evaluate infant faces even more positively (Lehmann *et al*., 2013). This experience-dependent response is also reflected in neural activation patterns (Leibenluft *et al*., 2004).

On a neural level, the perception of visual cuteness is associated with fast and specific responses in the orbitofrontal cortex (OFC) (Kringelbach *et al*., 2008). Those responses are assumed to function as a ‘rapid neural signal of parenting’ to initiate further caring behavior (Parsons *et al*., 2013). The engagement of the OFC coincides with parenting experience, and activation is stronger in response to signals from the own *vs* an unfamiliar infant (Nitschke *et al*., 2004; Strathearn *et al*., 2008).

The OFC is an essential hub in a network coding ‘pleasure’. This network furthermore includes the anterior insula, the ventral pallidum, the nucleus accumbens, the anterior cingulate cortex, the periaqueductal gray and the parabrachial nucleus (Berridge and Kringelbach, 2015; Kringelbach *et al*., 2016). Activation of the pleasure network, e.g. by cuteness perception, is associated with wanting, liking and reward learning. This provides the base for further motivational and affective responses in the context of parental care (Kringelbach *et al*., 2016).

Besides pleasure network (Noriuchi *et al*., 2008; Strathearn *et al*., 2008; Glocker *et al*., 2009), studies on visual cuteness revealed further areas responding to reward, namely, the ventral tegmentum area and the putamen (Noriuchi *et al*., 2008; Bos *et al*., 2018).

In their review, Kringelbach *et al*. (2016) proposed that infant cuteness is not only limited to vision but also evoked by auditory or olfactory stimuli. However, experimental studies applying cuteness to other senses are scarce. Huron (2005) reports that cute sounds, such as babbling or laughter, elicit responses linked to positive affect and caregiving. In line with that, infant noise evokes selective rapid responses in the periaqueductal gray of adults (Parsons *et al*., 2014) and different neural responses depending on parental status (Seifritz *et al*., 2003).

To the best of our knowledge, no studies to date investigate olfactory signals, which may similarly contribute to the perception of cuteness. This seems puzzling. Olfaction is one of the phylogenetically oldest sensory systems (Gottfried, 2006), and despite being considerably slower compared to visual or auditory cues, olfaction largely contributes to inter-individual communication in various species (Gillam, 2011). For many animals, olfactory identification of the offspring is crucial for survival (Kendrick, 1994). In humans, the maternal odor guides newborns toward the breast and soothes the child (Sullivan and Toubas, 1998; Nishitani *et al*., 2009). Conversely, mothers describe the body odor of their infant as a source of affection but also as an important informative cue guiding parental behavior (Okamoto *et al*., 2016).

Human chemosignals function in two aspects of this mutual bond (for a review, see Schäfer and Croy (2023)): first, corresponding to animal studies, body odors enable kin recognition between mother and infant (Porter *et al*., 1983; Cernoch and Porter, 1985). Second, parents prefer their child’s body odor over unfamiliar children’s odors (Schäfer *et al*., 2020a), which points to an inherent affective component transported by infantile chemosignals (Fleming *et al*., 1993). This preference links to relationship quality—i.e. mothers with postpartum bonding difficulties do not prefer their own child’s odor when compared to healthy mothers (Croy *et al*., 2019).

Previous research indicates that infantile body odors act in a fashion similar to the visual Kindchenschema: body odor composition changes over developmental stages (Grumbach, 2002); body odor evaluation depends on the developmental status of the sender (Schäfer *et al*., 2020b); and infantile odors are perceived as more positive than body odors of post-pubertal children (Weisfeld *et al*., 2003; Schäfer *et al*., 2020a).

Of the few studies targeting infantile chemosignals, most investigated behavior, while only three studies investigated neuronal responses. Two of those studies explored whether the neural correlates of infant body odor perception differ between mothers and nulliparae. They report either a general increased prefrontal cortex activity in mothers (Nishitani *et al*., 2014), or specific responses in striatal areas for both groups and slightly increased activations in mothers in the thalamus and the caudate nucleus (Lundström *et al*., 2013). Those results provide a first hint that infant body odors act as a rewarding cue. Our own study (Schäfer *et al*., 2019) aimed to optimize the signal-to-noise ratio of body odor presentation in the magnetic resonance imaging (MRI) scanner and thereby indicated a robust BOLD increase not only in primary and secondary olfactory processing areas but also in the superior temporal gyrus, a structure involved in social cognition (Schirmer, 2017) and infant face perception (Noriuchi *et al*., 2008).

While informative, the aforementioned studies are insufficient to support the idea of an olfactory Kindchenschema. According to Lorenz’s arguments, this requires a distinct and positive reaction to infants compared to older children, which is universal for both mothers and nulliparae. This reaction, based on cuteness perception, is processed in a neural network consisting of specific hubs that code pleasure and reward (Berridge and Kringelbach, 2015; Kringelbach *et al*., 2016) and thereby facilitate subsequent caring behavior.

The two studies presented here targeted the question of whether infantile body odors are, on a neural level, similarly processed to infantile faces, which in turn may support the idea of an olfactory ‘Kindchenschema’. We hypothesized that smelling body odors of infants and post-pubertal children leads to an increase in BOLD signal and functional connectivity in the networks of olfaction, pleasure and reward. We specifically hypothesized that activation and connectivity in pleasure and reward networks are (i) more pronounced after infant than post-pubertal body odor stimulation, (ii) related to experience and are more pronounced for mothers as compared to nulliparae and for the own *vs* another infant and (iii) positively related to the perception of body odors as being pleasant and rewarding. For the olfactory network, we expected no stimulus- or group-related differences. This was tested in two studies in which we recorded the neural response of a total of 78 women in the MRI scanner, while they smelled body odors of unfamiliar post-pubertal children, unfamiliar infants or their own infants.

## Methods

Both studies were conducted according to the ‘World Medical Association’s Declaration of Helsinki’ and approved by the University of Dresden ethics committee (Code: EK104032015). Written, informed consent was obtained from all subjects. The study—but not the specific neural hypothesis—was pre-registered at Deutsches Register klinischer Studien (DRKS, Code: DRKS00016539).

### Study I

Study I compared neural activations of mothers and nulliparae in response to an unfamiliar infantile *vs* post-pubertal body odor. We hypothesized that exposure to infantile body odor leads to an increase in BOLD signal and neural connectivity in olfactory areas and in areas related to pleasure and reward and that this pattern is more pronounced in response to infant body odor compared to post-pubertal body odor. We further expected greater BOLD signal and functional connectivity in the pleasure and reward areas in mothers compared to nulliparae. Finally, we hypothesized a positive relationship between perceptual ratings, BOLD signal, and functional connectivity strength.

#### Participants

Normosmic mothers (*N* = 20, *M*_age_ = 32.3 years, s.d. = 3.8 years) of a biological child (aged 0–3 years) and nulliparous women (*N* = 20, *M*_age_ = 24.2 years, s.d. = 1.2 years) smelled infantile and post-pubertal body odors in the MRI scanner. We sampled body odors from 25 infants (0–3 years) and 13 post-pubertal children (14–18 years), some related to the participating mothers. However, none of the mothers smelled the body odor of her child. There are no general guidelines about optimal sample sizes for olfactory functional MRI (fMRI) analysis yet; however, we based our sample estimation on previous studies in the field (Lundström *et al*., 2013; Schäfer *et al*., 2019). Further sample characteristics and demographic variables are presented in the Supplementary Material (Supplementary Table S7).

#### Inclusion and exclusion criteria

Recruitment of the participants was carried out using flyers and advertisements among university hospital staff, in mother–child courses (such as baby swimming course), as well as by personal invitations. After an initial contact, the inclusion and exclusion criteria were announced by e-mail and verified at the first appointment in the laboratory. Normosmic olfactory functioning of the participants was required for study inclusion and ensured by the Sniffin’ Sticks identification screening (Lötsch *et al*., 2016; Supplementary Table S7). Current pregnancy served as exclusion criteria for both groups. The age of infant body odor donors was set to 0–3 years—an age where robust olfactory parental kin recognition and preference are observed (Schäfer *et al*., 2020a) and which is well below the first major development of puberty and adrenarche, starting at the age of ∼6 years (Dorn *et al*., 2006). The post-pubertal body odor donors’ age was 14–18 years to ensure main pubertal hormonal transitions (Dorn *et al*., 2006). Exclusion criteria for all body odor donors were disability or chronic disease.

#### Study procedure

Participants received the study kit for body odor sampling at home during the initial laboratory visit. After sampling the body odors with a standardized protocol (Supplementary Material), we conducted the experimental fMRI session.

#### Experimental design

Mothers smelled body odor samples of an unfamiliar infant [sex- and age-matched to their infant (average age match: 2.63 months and maximum difference: 7 months)] and of an unfamiliar sex-matched child of post-pubertal age. Sex- matching was performed to exclude sex as a potential source of familiarity and was successfully performed in all but one mother. This mother was presented with a male infant body odor (sex-matched to her own child) and a female post-pubertal body odor. All nulliparous women smelled the body odors of an infant and a sex-matched post-pubertal child.

The infant and post-pubertal body odor were presented twice each in randomized order (four runs total, 6 min each) by an air dilution, computer-controlled olfactometer (Sommer *et al*., 2012), which was positioned next to the scanning room and delivered a constant airstream of 2 l/min via 5 m Teflon tubes to the participant’s nostrils (birhinal presentation, inner diameter of nose piece and tube 4 mm).

**Fig. 1.**
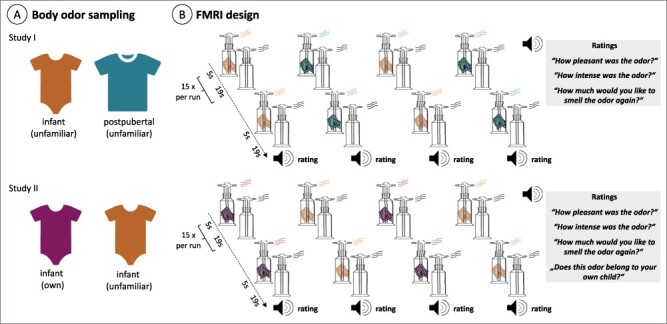
Study procedure. (A) Body odors were sampled from unfamiliar infants and post-pubertal children in study 1 and from the own and unfamiliar infants in study II (see ‘Methods’ section for details). Those odors were presented to mothers and nulliparae (study 1) or mothers only (study II) within the MRI scanner (B). Each participant received 4 runs and 15 stimulation periods [5 s ON (body odor presentation) and 19 s OFF (clean air presentation)], adapted after the protocol of Schäfer et al., (2019): The design matters: how to detect neural correlates of baby body odors. Frontiers in Neurology, 9, 1182. After each run, the participants were asked to evaluate pleasantness, intensity and wanting (from 0—‘not at all’ to 100 ‘very’) of the presented odor. In study 2, mothers were additionally asked to state whether the presented odor belonged to the own child (‘yes’ or ‘no’).

During each run, the odor was presented with an optimized design of 15 repetitions of 5 s continuous stimulation followed by a 19 s baseline with clean air (Schäfer *et al*., 2019). After each run, the participants were asked via headphones to verbally rate pleasantness (‘How pleasant was the odor?’), intensity (‘How intense was the odor?’) and wanting (‘How much would you like to smell the odor again?’) of the stimulus on a scale ranging from 0 to 100 (e.g. ‘not pleasant at all’ to ‘very pleasant’) (Figure 1; for details, see Supplementary Table S1 and Supplementary Figure S1).

Participants were instructed to breathe regularly through the nose. As sniffing patterns vary between individuals and often depend on the specific task (Beauchamp *et al*., 2014), we guided breathing by a presentation displayed to the participants in the scanner. This presentation showed a traffic light color coding indicating inhalation to start in 2 s (yellow) or now (green). The procedure was explained and practiced prior to the experiment.

#### MRI procedures

Imaging data were acquired on a Siemens 3 T Prisma scanner by a T2*-weighted gradient-echo, echo-planar imaging sequence (repitition time (TR) = 2.5 s, echo time (TE) = 30 ms, flip angle 90°, 25 mm × 1 mm axial slices, 3.6 × 3.6 in-plane resolution) using a 32-channel head coil. Precise anatomical mapping of the functional data was enabled by adding a high-resolution T1 sequence (TR = 2.3 s, TE = 3.43 ms, 0.7 × 1 mm in-plane resolution). As a parallel imaging technique, Generalized Autocalibrating Partial Parallel Acquisition (acceleration factor = 2) was used. Orientation of the scanning planes was parallel to the anterior–posterior commissure line, and according to our regions of interest (ROIs), we sampled from the cerebellum up to the dorsal end of the cingulate cortex. In total, 150 volumes were acquired per session in an interleaved fashion.

#### Data analyses

##### Regions of interest (ROIs).

**Fig. 2.**
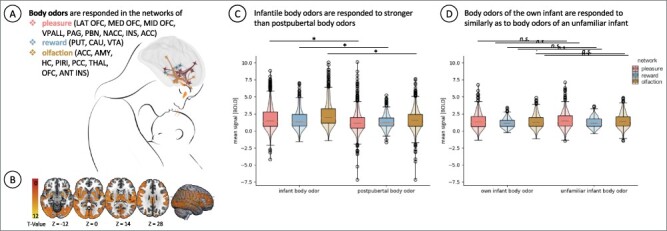
BOLD signal change after smelling infantile and post-pubertal body odors. (A) Investigated networks with the respective ROIs are visualized (pleasure: LAT OFC = lateral orbitofrontal cortex, MED OFC = medial orbitofrontal cortex, MID OFC = mid-anterior orbitofrontal cortex, VPALL = ventral pallidum, PAG = periaqueductal gray, PBN = parabrachial nucleus, NACC = nucleus accumbens, INS = insula, ACC = anterior cingulate cortex; reward: PUT = putamen, CAU = caudate, VTA = ventral tegmentum area; olfaction: ACC = anterior cingulate cortex, AMY = amygdala, HC = hippocampus, PIRI = piriform cortex, PCC = posterior cingulate cortex, THAL = thalamus, OFC, ANT INS = anterior insula). (B) Whole-brain analysis of the infant body odor condition *vs* baseline (study I) shows a significant BOLD signal increase over multiple brain areas, encompassing the pleasure, reward and olfaction network (height threshold: *P *< 0.05, FWE corrected). The other conditions of study I and study II revealed similar activations and are depicted in the Supplementary Material, Figure S2. (C) BOLD signal extracted values plotted per network and condition show higher (*P < 0.05) signal strength after smelling infantile *vs* post-pubertal body odors in each of the networks (study I). (D) BOLD signal extracted values plotted per network and condition show no significant (n.s.) difference between smelling the body odors of the own *vs* another infant (study II).

We constructed the ROIs of the olfactory network (amygdala, piriform cortex, thalamus, hippocampus, posterior cingulate cortex, OFC, anterior cingulate cortex and insula), the pleasure network (lateral OFC, mid-anterior OFC, medial OFC, nucleus accumbens and ventral pallidum) and the reward network (periaqueductal gray, parabrachial nucleus, ventral tegmental area, caudate and putamen) with the WFU PickAtlas 3.0.3 toolbox (Maldjian *et al*., 2003) for Statistical Parametric Mapping (SPM) and used the automated anatomic labeling (AAL), individual brain atlases using SPM and ascending arousal network Atlas (Alemán-Gómez, 2006; Edlow *et al*., 2012; Rolls *et al*., 2020). The superior temporal gyrus (STG) was analyzed as an additional ROI based on previous findings (Schäfer *et al*., 2019). Detailed results on the STG are presented in the Supplementary Material. ROI definitions are displayed in see Figure 2A and Supplementary Table S3. Furthermore, guided by previous findings (Swain, 2011), we also investigated the STG. Those results are presented in the Supplementary Material.

##### BOLD signal analysis.

The functional images were pre-processed after a standardized procedure implemented in SPM 12 (Wellcome Trust Center for Neuroimaging, London, UK, implemented in MATLAB R2017b, MathWorks, Inc., Natick, MA, USA). Details are reported in the Supplementary Material.

###### Statistical analysis.

We first contrasted odor stimulation periods, for each run separately, to the subsequent baselines using SPM 12 (first-level analysis). For the SPM 12 second-level analyses, we calculated *t*-tests thresholded at p family-wise error rate <0.05: infantile body odor *vs* baseline and post-pubertal body odor *vs* baseline. This was done for the whole group of participants and separately for both nulliparae and mothers (for whole-brain results, see Figure 2B, supplementary Table S8 and Supplementary Figure S2). This analysis revealed a significant odor-induced activation within each of the pre-defined ROIs.

To further analyze the three hypothesized networks, we first created a 4 mm sphere around the peak voxel of each ROI (Supplementary Table 3) and extracted the mean beta signals within each sphere per run for each subject using MarsBar (Brett *et al*., 2002).

Coherently, the averaged BOLD signals exceeded the chance level for each ROI (one-sample *t*-test, *P* ≤ 0.001, *t* = 6.47 to 16.01). After that, the extracted BOLD signal was entered as a target in Generalized linear mixed models (GLMM)s computed in SPSS (IBM Corp. Released 2020. IBM SPSS Statistics for Windows, Version 27.0. Armonk, NY: IBM Corp). We calculated one GLMM per network. Each participant served as individual within the model, each condition, run and ROI served as repeated measure levels. As model specifications for the GLMMs we used robust estimations and Kenward–Roger approximation. The identity function was used as the linking function according to the linear model specifications. The following effects were modeled: main effect of condition (infant *vs* post-pubertal odor) and main effect of group (mothers *vs* nulliparae). *Post hoc* analyses were conducted using *t*-test pairwise comparisons. To capture the relation between the hedonic percept and neural activity, repeated measurement correlations were calculated in R (v4.1.2; R Core Team 2021). between ratings of ‘wanting to smell the odor again’ and pleasantness and mean extracted signal per network. Only significant correlations are reported. All statistical tests were calculated two-sided.

##### Functional connectivity analysis.

Functional connectivity analysis was performed with the CONN toolbox release 19c (Whitfield-Gabrieli and Nieto-Castanon, 2012; Nieto-Castanon, 2020), which runs under MATLAB (The MathsWorks Inc., Natick, MA, USA). The functional images were pre-processed after a standardized pre-processing procedure implemented in the CONN toolbox (Nieto-Castanon, 2020; Supplementary Material).

###### Statistical analysis.

First- and second-level ROI-to-ROI functional connectivity analysis was calculated based on the CONN-implemented GLM over all pre-defined networks and separately for each network and condition. Further, the between-condition contrast infantile body odor > post-pubertal body odor was analyzed for (i) the whole group of participants, (ii) nulliparae and (iii) mothers, again over all networks and for each of the pre-defined networks. Then, perceptual ratings were included as covariates in the same connectivity analyses. The results of the functional connectivity analysis are presented false discovery rate (FDR)-corrected, thresholded at *P* < 0.05.

### Study II

Study II compared neural activations in response to the body odor of the own and an unfamiliar infant in a sample of mothers. We hypothesized that exposure to infantile body odor leads to increased BOLD signal and neural connectivity in olfactory areas and in areas related to pleasure and reward as well as the STG and that this pattern is more pronounced for the body odor of one’s own compared to an unfamiliar infant. We further hypothesized a positive relation between perceptual ratings and BOLD signal as well as functional connectivity strength.

#### Participants

Normosmic mothers (*N* = 38, *M*_age_ = 32.1 years, s.d. = 3.1 years) with a child between 0 and 3 years participated in the study (Supplementary Table S7). There was no overlap of participants or body odor donors between study I and study II. The infantile body odors served as own child for the own mother and as unfamiliar samples for the other participants. Inclusion and exclusion criteria were the same as in study I.

#### Experimental design

Study II was conducted with the same odor donation and presentation specifications as reported in study I with the following exceptions: each participant was presented with body odor samples of their own and an age- and sex-matched unfamiliar infant (average age match: 2.17 months with a maximum difference of 6 months). In addition to the three perceptual ratings after the body odor presentation, the mothers were asked to state whether the presented odor belonged to their child (‘yes/no’).

#### BOLD extraction analyses

Similar to study I, data were pre-processed, and first- and second-level statistics were calculated in SPM (compare Supplementary S8 for whole-brain results). Individual BOLD signal estimates were extracted per participant and run for each of the 4 mm spheres reported in Supplementary Table S3. One-sample *t*-tests were computed for each network, and a GLMM (same specifications as in study I) was analyzed, where the main effect of condition (own *vs* unfamiliar infant body odor) was modeled. In the next step, the main effect of correct identification on the mean signal was included in the GLMM.

To capture the relation between the hedonic perception and neural activity, repeated measurement correlations were calculated between ratings of ‘wanting to smell the odor again’ and pleasantness and mean extracted signal per network. Only significant correlations are reported. All statistical tests were two-sided calculations.

#### Functional connectivity analyses

The functional data were processed according to the processing pipeline implemented in study I. First- and second-level ROI-to-ROI functional connectivity analysis was calculated based on a GLM-weighted model separately for each network. The overall connectivity of the infant body odor as well as the between-condition contrast own infant body odor > unfamiliar infant body odor was calculated over all subjects and for each network. In the last step, perceptual ratings were included as covariates in the same connectivity analyses. In line with study I, ROI-to-ROI connectivity values were extracted and then entered as a target in a GLMM with the same specifications as described above in SPSS to assess the effect of condition on the overall connectivity per network.

## Results

### Infant body odors lead to an increase in BOLD signal within the reward, pleasure and olfactory network

All body odor stimuli elicited significant above-threshold activations in each ROI of each of the three networks (Figure S3), with the single exception of the parabrachial nucleus in study II. Neural responses were comparable between study I and study II (compare Figure 2C and D, Supplementary Figure S2, S3 and Supplementary Table S4). The effect size of the difference between body odor stimulation *vs* baseline was very high for all networks ranging between Cohen’s *d* 1.6 and 2.5.

In line with the hypothesis, smelling the body odor of infants as compared to post-pubertals led to a significantly larger BOLD signal increase in the reward and in the pleasure network (reward network: *t*(192) = 3.34, *P* = 0.001, *d* = 0.27, pleasure network: *t*(762) = 5.99, *P* < 0.001, *d* = 0.30, Figure 2C and Supplementary Table S4). The same pattern was observed for the olfactory network (*t*(804) = 7.07, *P* < 0.001, *d* = 0.45). Aligning with our assumption, the BOLD signal increase within the olfactory network did not differ between mothers and nulliparae or between the own and another infant. In contrast to our expectation, there was also no difference for the reward and pleasure network (Figure 2D and Supplementary Table S4). Correct recognition of the own child aligned with the BOLD signal increase in the pleasure network (*F*(2502) = 3.07, *P* = 0.048; Supplementary Table S5).

The wish to smell the odor again related positively to BOLD signal increase in the pleasure network. However, this relation was weak and only significant in study I: (study I: *r* = 0.06, *P* = 0.029; study II: *r* = −0.01, *P* = 0.597; Supplementary Table S5).

### Infant body odors lead to enhanced functional connectivity

**Fig. 3.**
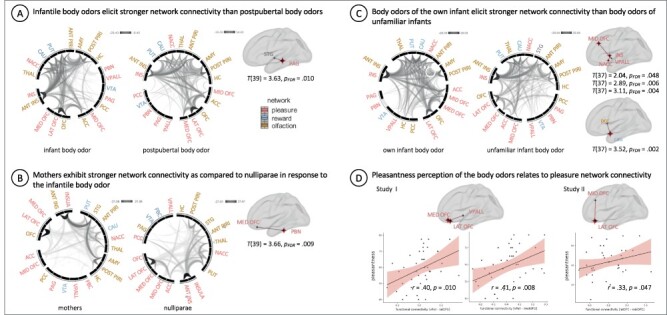
Functional network connectivity after smelling infantile and post-pubertal body odors. (A) Study I. Functional network connectivity between all nodes of interest [pleasure, reward, olfaction, social cognition (superior temporal gyrus)] plotted across all subjects for the infant body odor condition and the post-pubertal body odor condition. The contrast between the conditions revealed a significant main effect showing increased connectivity in response to the infantile *vs* the post-pubertal body odor in the depicted pleasure and social cognition nodes, displayed with the respective *T*-value. (B) Study I: Functional network connectivity between all nodes of interest plotted per group (mothers and nulliparae) for the infant body odor condition. The contrast between the groups revealed a significant main effect of group showing increased connectivity in response to the infantile body odor for mothers compared to nulliparae between the depicted pleasure nodes, displayed with the respective *T*-value. (C) Study II: Functional network connectivity between all nodes of interest plotted for the own and the unfamiliar infant body odor condition. The contrast between the conditions revealed a significant main effect showing increased connectivity in response to the own *vs* the unfamiliar infantile body odor between the depicted pleasure and reward nodes, displayed with the respective *T*-values. (D) Correlational analyses between perceptual ratings and functional connectivity across conditions revealed significant positive associations between pleasantness ratings and functional connectivity between pleasure nodes, as plotted per study with regression line and the respective standard deviation. All results are displayed *P* < 0.05, FDR-corrected. Abbreviations: pleasure: LAT OFC = lateral orbitofrontal cortex, MED OFC = medial orbitofrontal cortex, MID OFC = mid-anterior orbitofrontal cortex, VPALL = ventral pallidum, PAG = periaqueductal gray, PBN = parabrachial nucleus, NACC = nucleus accumbens, INS = insula, ACC = anterior cingulate cortex; reward: PUT = putamen, CAU = caudate, VTA = ventral tegmentum area; olfaction: ACC = anterior cingulate cortex, AMY = amygdala, HC = hippocampus, ANT PIRI = anterior piriform cortex, POST PIRI = posterior piriform cortex, PCC = posterior cingulate cortex, THAL = thalamus, OFC, ANT INS = anterior insula; social cognition: STG).

All body odor stimuli of study I and II elicited increased temporal alignment in each of the three networks, with very few exceptions (Figure 3A, C, Supplementary Materials 1–8). In line with to our hypotheses, across all participants, FDR-corrected connectivity within the pleasure nodes was stronger for infantile than post-pubertal body odors, namely STG to periaqueductal gray (*T* = 3.63, Supplementary Table S6 and Figure 3A). The reverse contrast revealed no significant effect. Hence, there was no enhanced connectivity after smelling post-pubertal as opposed to infantile odors.

Supporting our hypotheses on maternal experience, FDR-corrected functional connectivity was stronger in mothers than in nulliparae (medial OFC to parabrachial nucleus, *T* = 3.66, Table S6, Figure 3B) and for the own *vs* another infant (caudate to posterior cingulate cortex; ventral pallidum to mid-anterior OFC, to insula and to nucleus accumbens, *T* = 2.04–3.52, Supplementary Table S6 and Figure 3C). The reverse contrasts revealed no significant effect. According to our expectation, no effect of body odor donor or receiver was observed for the olfactory network connectivity.

Pleasantness ratings related positively to pleasure network connectivity in both studies (study I: ventral pallidum to medial OFC, *r *= 0.41, *P* = 0.008, and ventral pallidum to lateral OFC, *r* = 0.40, *P* = 0.010; *n* = 40; study II: lateral OFC to mid-anterior OFC, *r* = 0.33, *P* = 0.047, *n* = 38; Supplementary Table S6 and Figure 3D).

## Discussion

Our results demonstrate that infant body odors enhance BOLD signal and functional connectivity within olfactory, pleasure and reward networks. This neural signature is stronger for infantile than post-pubertal body odors, suggesting that infant body odors function as a specifically rewarding cue and are processed in a similar fashion to infant faces. In the following, we discuss the results in detail.

The elevated BOLD signal in response to infantile odors aligns with previous findings of increased OFC reactivity when viewing infant *vs* adult faces (Kringelbach *et al*., 2008). This is not solely explained by shared hubs (insula and OFC are hubs of the pleasure and the olfactory network) but was observed in all olfactory areas. We propose two alternative explanations: First, infantile chemosignals may trigger an elevated activation pattern in the glomeruli of the olfactory bulb, which initiates increased processing in primary and secondary olfactory areas. Second, the recognition of the body odor as “infant” may increase its salience and trigger feedback loops reflected in augmented processing in primary and secondary olfactory areas. The amygdala with its close interlink to the salience network (Seeley, 2019) may particularly contribute here.

The increased BOLD signal was paralleled by stronger connectivity between the STG and the periaqueductal gray, which may indicate a pathway for prioritized social processing that guides adaptive behavior. The STG is not only involved in smelling infant body odors (Swain, 2011) but also a hub for social perception (Swain, 2011). The periaqueductal gray is a pleasure network structure, involved in prioritizing maternal behavior, as demonstrated in female nursing rats (Sukikara *et al*., 2006). Notably, co-activation of both structures has been observed in response to auditory cuteness (Parsons *et al*., 2014) and infant’s face perception in mothers (Noriuchi *et al*., 2008).

The results are supported by a correlation between the BOLD activation and connectivity within the pleasure network and positive odor perception. Specifically, the observed correlation between body odor liking and OFC involvement here in this study fits previous results, which demonstrated that OFC activation relates to maternal ratings of mood and warm feelings while viewing infant faces (Nitschke *et al*., 2004).

Experience did not relate to neural activation patterns but to neural connectivity, suggesting a more efficient interplay of pleasant network structures. Maternal experience was associated with enhanced functional connectivity between the medial OFC and the parabrachial nucleus. The medial part of the OFC controls learning, memory and valence associated with incentive stimuli (Berridge and Kringelbach, 2015). The parabrachial nucleus as ‘a brainstem mechanism for pleasure’ (Berridge and Kringelbach, 2015) guides motivational and emotional processes (Damasio, 2010; Wu *et al*., 2012). The increased interplay of those structures may hence reflect an experience-related effective integration of pleasure coding, promoting subsequent motivational responses in the context of caregiving.

Familiarity of the mother with her own infant’s body odor linked to increased functional connectivity from the ventral pallidum to the nucleus accumbens, the anterior insula and the mid-anterior OFC. The ventral pallidum is considered a ‘hedonic hotspot’ (Berridge and Kringelbach, 2015) and is strongly targeted by projections from the nucleus accumbens. Both areas build a functional bridge for opioid-stimulated liking reactions (Smith and Berridge, 2007).

The anterior insula is a critical hub in the salience network (Seeley, 2019), and the mid-anterior OFC is part of the pleasure network that encodes valence (Jensen *et al*., 2007). The connection between the insula and the ventral pallidum initiates salience network activity, while the connection between ventral pallidum and OFC promotes the encoding of such cues, facilitating subsequent motivational responses to the highly salient and familiar odor of the own child.

Taken together, we postulate that our studies provide evidence toward an extension of the Kindchenschema—from an infant’s face to an infant’s scent—hence, infantile body odors may transport olfactory cuteness. To critically test this assumption, future studies may compare the selective processing of infantile stimuli in the visual and olfactory domain in the same perceivers. Such studies may also include subjective ratings of cuteness.

In addition, our data demonstrate neural patterns in response to infantile chemosignals that exhibit similarities to the ‘parental brain’ (Swain, 2011). According to that model, the infant cue—hence, the infant body odor—activates cognitive (e.g. OFC and STG) and emotional (e.g. insula, amygdala and striatum) nodes important for empathy and motivation, which jointly shape the output of parental behaviors. Following from this model, the observed activation patterns after smelling the infant prepare the adult brain to initiate caregiving and empathetic responses. This aligns with prior research demonstrating that infant body odors are generally perceived as pleasant (Schäfer *et al*., 2020a) and contribute to positive feelings (Fleming *et al*., 1993). This affective component of the infant’s body odor may in turn promote physical closeness to the child and thus contribute to other affiliative behaviors, such as affective touch, that, in turn, reinforce bonding behavior.

In that sense, further studies are warranted linking the neural responses to maternal affect and actual attachment quality, e.g. as demonstrated in neuroimaging studies on visual cues (Lowell *et al*., 2023). Considering the generalizability of the results, it has to be regarded that our studies only included healthy women with an intact sense of smell, and the group of nulliparae consisted of a relatively homogenous student population. However, we do not assume that odor processing fundamentally differs in other populations. While the beneficial impact of maternal chemosignals on adult–infant brain synchrony has been demonstrated (Endevelt-Shapira *et al*., 2021), similar studies using infant chemosignals are pending.

## Conclusion

Our results suggest that an infant body odor, similar to an infant face, functions as an appealing social cue related to cuteness by eliciting BOLD responses of pleasure and reward in mothers as well as nulliparae. We further showed that functional connectivity between central nodes of the pleasure and reward network increased in relation to kin and maternal state. Taken together, our findings highlight the relevance of infantile chemosignals in preparing the maternal brain to perceive affection and to respond with care.

## Supplementary Material

nsae038_Supp

## Data Availability

The datasets supporting this article have been uploaded as part of the electronic supplementary material. Extracted functional connectivity raw values are provided in Supplementary Materials 1–4, and cluster exports of the functional connectivity analyses are provided in Supplementary Materials 5–8.

## References

[R1] Alemán-Gómez Y. (2006). IBASPM: toolbox for automatic parcellation of brain structures. In: 12th Annual Meeting of the Organization for Human Brain Mapping. June 11–15, 2006, Florence Italy.

[R2] Beauchamp J., Scheibe M., Hummel T., et al. (2014). Intranasal odorant concentrations in relation to sniff behavior. *Chemistry and Biodiversity*, 11(4), 619–38. 24706630 10.1002/cbdv.201300320

[R3] Berridge K.C., Kringelbach M.L. (2015). Pleasure systems in the brain. *Neuron*, 86(3), 646–64. 25950633 10.1016/j.neuron.2015.02.018 PMC4425246

[R4] Bos P.A., Spencer H., Montoya E.R. (2018). Oxytocin reduces neural activation in response to infant faces in nulliparous young women. *Social Cognitive & Affective Neuroscience*, 13(10), 1099–109. 30203082 10.1093/scan/nsy080 PMC6204485

[R5] Brett M. , et al. (2002). Region of interest analysis using an SPM toolbox. In: 8th International Conference on Functional Mapping of the Human Brain. Sendai.

[R6] Brosch T., Sander D., Scherer K.R. (2007). That baby caught my eye… attention capture by infant faces. *Emotion*, 7(3), 685–9. 17683225 10.1037/1528-3542.7.3.685

[R7] Cernoch J.M., Porter R.H. (1985). Recognition of maternal axillary odors by infants. *Child Development*, 56(6), 1593–8. 4075877

[R8] Croy I., Mohr T., Weidner K., et al. (2019). Mother-child bonding is associated with the maternal perception of the child’s body odor. *Physiology and Behavior*, 198, 151–7. 30261171 10.1016/j.physbeh.2018.09.014

[R9] Damasio A. (2010). *Self Comes to Mind: Constructing the Conscious Brain by Antonio Damasio*. New York City: Pantheon.

[R10] Dorn L.D., Dahl R.E., Woodward H.R., et al. (2006). Defining the boundaries of early adolescence: a user’s guide to assessing pubertal status and pubertal timing in research with adolescents. *Applied Developmental Science*, 10(1), 30–56.

[R11] Edlow B.L., Takahashi E., Wu O., et al. (2012). Neuroanatomic connectivity of the human ascending arousal system critical to consciousness and its disorders. *Journal of Neuropathology and Experimental Neurology*, 71(6), 531–46. 22592840 10.1097/NEN.0b013e3182588293 PMC3387430

[R12] Endevelt-Shapira Y., Djalovski A., Dumas G., et al. (2021). Maternal chemosignals enhance infant-adult brain-to-brain synchrony. *Science Advances*, 7(50), 1–11. 10.1126/sciadv.abg6867 PMC8664266 34890230

[R13] Fleming A.S., Corter C., Franks P., et al. (1993). Postpartum factors related to mother’s attraction to newborn infant odors. *Developmental Psychobiology: The Journal of the International Society for Developmental Psychobiology*, 26(2), 115–32. 10.1002/dev.420260204 8467961

[R14] Gillam E. (2011). An introduction to animal communication. *Nature Education*, 3(10), 70.

[R15] Glocker M.L., Langleben D.D., Ruparel K., et al. (2009). Baby schema in infant faces induces cuteness perception and motivation for caretaking in adults. *Ethology*, 115(3), 257–63. 22267884 10.1111/j.1439-0310.2008.01603.x PMC3260535

[R16] Gottfried J.A. (2006). Smell: central nervous processing. *Taste and Smell* 63, 44–69. 10.1159/000093750 16733332

[R17] Grumbach M.M. (2002). The neuroendocrinology of human puberty revisited. *Hormone Research in Paediatrics*, 57(Suppl. 2), 2–14. 10.1159/000058094 12065920

[R18] Huron D. (2005). The plural pleasures of music. In: *Proceedings of the 2004 Music and Science Conference*, Stockholm.

[R19] Jensen J., Smith A.J., Willeit M., et al. (2007). Separate brain regions code for salience vs. valence during reward prediction in humans. *Human Brain Mapping*, 28(4), 294–302. 16779798 10.1002/hbm.20274 PMC6871333

[R20] Kendrick K.M. (1994). Neurobiological correlates of visual and olfactory recognition in sheep. *Behavioural Processes*, 33(1), 89–111. 24925241 10.1016/0376-6357(94)90061-2

[R21] Kringelbach M.L., Lehtonen A., Squire S., et al. (2008). A specific and rapid neural signature for parental instinct. *PLoS One*, 3(2), e1664. 10.1371/journal.pone.0001664 PMC2244707 18301742

[R22] Kringelbach M.L., Stark E.A., Alexander C., et al. (2016). On cuteness: unlocking the parental brain and beyond. *Trends in Cognitive Sciences*, 20(7), 545–58. 27211583 10.1016/j.tics.2016.05.003 PMC4956347

[R23] Lehmann V., Huis In‘t Veld E.M.J., Vingerhoets A.J.J.M. (2013). The human and animal baby schema effect: correlates of individual differences. *Behavioural Processes*, 94, 99–108. 23353724 10.1016/j.beproc.2013.01.001

[R24] Leibenluft E., Gobbini M.I., Harrison T., et al. (2004). Mothers’ neural activation in response to pictures of their children and other children. *Biological Psychiatry*, 56(4), 225–32. 15312809 10.1016/j.biopsych.2004.05.017

[R25] Lorenz K. (1943). Die angeborenen Formen möglicher Erfahrung. *Zeitschrift Für Tierpsychologie*, 5(2), 235–409.

[R26] Lötsch J., Ultsch A., Hummel T. (2016). How many and which odor identification items are needed to establish normal olfactory function? *Chemical Senses*, 41(4), 339–44. 26857742 10.1093/chemse/bjw006

[R27] Lowell A.F., Dell J., Potenza M.N., et al. (2023). Adult attachment is related to maternal neural response to infant cues: an ERP study. *Attachment & Human Development*, 25(1), 71–88. 33522435 10.1080/14616734.2021.1880057 PMC10861024

[R28] Lundström J.N., Mathe A., Schaal B., et al. (2013). Maternal status regulates cortical responses to the body odor of newborns. *Frontiers in Psychology*, 4, 1–6. 24046759 10.3389/fpsyg.2013.00597 PMC3763193

[R29] Maldjian J.A., Laurienti P.J., Kraft R.A., et al. (2003). An automated method for neuroanatomic and cytoarchitectonic atlas-based interrogation of fMRI data sets. *Neuroimage*, 19(3), 1233–9. 12880848 10.1016/s1053-8119(03)00169-1

[R30] Nieto-Castanon A. (2020). *Handbook of Functional Connectivity Magnetic Resonance Imaging Methods in CONN*. Boston: Hilbert Press.

[R31] Nishitani S., Kuwamoto S., Takahira A., et al. (2014). Maternal prefrontal cortex activation by newborn infant odors. *Chemical Senses*, 39(3), 195–202. 24403536 10.1093/chemse/bjt068

[R32] Nishitani S., Miyamura T., Tagawa M., et al. (2009). The calming effect of a maternal breast milk odor on the human newborn infant. *Neuroscience Research*, 63(1), 66–71. 19010360 10.1016/j.neures.2008.10.007

[R33] Nitschke J.B., Nelson E.E., Rusch B.D., et al. (2004). Orbitofrontal cortex tracks positive mood in mothers viewing pictures of their newborn infants. *Neuroimage*, 21(2), 583–92. 14980560 10.1016/j.neuroimage.2003.10.005

[R34] Noriuchi M., Kikuchi Y., Senoo A. (2008). The functional neuroanatomy of maternal love: mother’s response to infant’s attachment behaviors. *Biological Psychiatry*, 63(4), 415–23. 17686467 10.1016/j.biopsych.2007.05.018

[R35] Okamoto M., Shirasu M., Fujita R., et al. (2016). Child odors and parenting: a survey examination of the role of odor in child-rearing. *PLoS One*, 11(5), e0154392. 10.1371/journal.pone.0154392 PMC4854394 27138751

[R36] Parsons C.E., Stark E.A., Young K.S., et al. (2013). Understanding the human parental brain: a critical role of the orbitofrontal cortex. *Social Neuroscience*, 8(6), 525–43. 24171901 10.1080/17470919.2013.842610

[R37] Parsons C.E., Young K.S., Joensson M., et al. (2014). Ready for action: a role for the human midbrain in responding to infant vocalizations. *Social Cognitive & Affective Neuroscience*, 9(7), 977–84. 23720574 10.1093/scan/nst076 PMC4090964

[R38] Parsons C.E., Young K.S., Kumari N., et al. (2011). The motivational salience of infant faces is similar for men and women. *PLoS One*, 6(5), e20632. 10.1371/journal.pone.0020632 PMC3105111 21655195

[R39] Porter R.H., Cernoch J.M., McLaughlin F.J. (1983). Maternal recognition of neonates through olfactory cues. *Physiology and Behavior*, 30(1), 151–4. 6836038 10.1016/0031-9384(83)90051-3

[R0045a] R Core Team (2021). R: A language and environment for statistical computing. *R Foundation for Statistical Computing*, Vienna, Austria.

[R40] Rolls E.T., Huang -C.-C., Lin C.-P., et al. (2020). Automated anatomical labelling atlas 3. *Neuroimage*, 206, 116189. 10.1016/j.neuroimage.2019.116189 31521825

[R41] Schäfer L., Croy I. (2023). An integrative review: human chemosensory communication in the parent-child relationship. *Neuroscience and Biobehavioral Reviews* 153, 105336. 10.1016/j.neubiorev.2023.105336 37527693

[R42] Schäfer L., Hummel T., Croy I. (2019). The design matters: how to detect neural correlates of baby body odors. *Frontiers in Neurology*, 9, 1–11. 10.3389/fneur.2018.01182 PMC6343458 30700979

[R43] Schäfer L., Sorokowska A., Sauter J., et al. (2020a). Body odours as a chemosignal in the mother–child relationship: new insights based on an human leucocyte antigen-genotyped family cohort. *Philosophical Transactions of the Royal Society B: Biological Sciences*, 375(1800), 20190266. 10.1098/rstb.2019.0266 PMC7209942 32306871

[R44] Schäfer L., Sorokowska A., Weidner K., et al. (2020b). Children’s body odors: hints to the development status. *Frontiers in Psychology*, 11, 1–9. 32194481 10.3389/fpsyg.2020.00320 PMC7064733

[R45] Schirmer A. (2017). Is the voice an auditory face? An ALE meta-analysis comparing vocal and facial emotion processing. *Social Cognitive & Affective Neuroscience*, 13(1), 1–13. 10.1093/scan/nsx142 PMC5793823 29186621

[R46] Seeley W.W. (2019). The salience network: a neural system for perceiving and responding to homeostatic demands. *The Journal of Neuroscience*, 39(50), 9878–82. 31676604 10.1523/JNEUROSCI.1138-17.2019 PMC6978945

[R47] Seifritz E., Esposito F., Neuhoff J.G., et al. (2003). Differential sex-independent amygdala response to infant crying and laughing in parents versus nonparents. *Biological Psychiatry*, 54(12), 1367–75. 14675800 10.1016/s0006-3223(03)00697-8

[R48] Senese V.P., De Falco S., Bornstein M.H., et al. (2013). Human infant faces provoke implicit positive affective responses in parents and non-parents alike. *PLoS One*, 8(11), e80379. 10.1371/journal.pone.0080379 PMC3840010 24282537

[R49] Smith K.S., Berridge K.C. (2007). Opioid limbic circuit for reward: interaction between hedonic hotspots of nucleus accumbens and ventral pallidum. *The Journal of Neuroscience*, 27(7), 1594–605. 17301168 10.1523/JNEUROSCI.4205-06.2007 PMC6673729

[R50] Sommer J.U., Maboshe W., Griebe M., et al. (2012). A mobile olfactometer for fMRI-studies. *Journal of Neuroscience Methods*, 209(1), 189–94. 22683953 10.1016/j.jneumeth.2012.05.026

[R51] Strathearn L., Li J., Fonagy P., et al. (2008). What’s in a smile? Maternal brain responses to infant facial cues. *Pediatrics*, 122(1), 40–51. 18595985 10.1542/peds.2007-1566 PMC2597649

[R52] Sukikara M.H., Mota-Ortiz S.R., Baldo M.V., et al. (2006). A role for the periaqueductal gray in switching adaptive behavioral responses. *The Journal of Neuroscience*, 26(9), 2583–9. 16510737 10.1523/JNEUROSCI.4279-05.2006 PMC6793662

[R53] Sullivan R.M., Toubas P. (1998). Clinical usefulness of maternal odor in newborns: soothing and feeding preparatory responses. *Biology Neonate*, 74(6), 402–8. 10.1159/000014061 PMC2046216 9784631

[R54] Swain J.E. (2011). The human parental brain: in vivo neuroimaging. *Progress in Neuro-Psychopharmacology and Biological Psychiatry*, 35(5), 1242–54. 21036196 10.1016/j.pnpbp.2010.10.017 PMC4329016

[R55] Tinbergen N. (1963). On aims and methods of ethology. *Zeitschrift Für Tierpsychologie*, 20(4), 410–33.

[R56] Weisfeld G.E., Czilli T., Phillips K.A., et al. (2003). Possible olfaction-based mechanisms in human kin recognition and inbreeding avoidance. *Journal of Experimental Child Psychology*, 85(3), 279–95. 12810039 10.1016/s0022-0965(03)00061-4

[R57] Whitfield-Gabrieli S., Nieto-Castanon A. (2012). Conn: a functional connectivity toolbox for correlated and anticorrelated brain networks. *Brain Connectivity*, 2(3), 125–41. 22642651 10.1089/brain.2012.0073

[R58] Wu Q., Clark M.S., Palmiter R.D. (2012). Deciphering a neuronal circuit that mediates appetite. *Nature*, 483(7391), 594–7. 22419158 10.1038/nature10899 PMC4000532

