# Supplementary material for: The scent of cuteness—neural signatures of infant body odors
Source: Soc Cogn Affect Neurosci. 2024 Jun 8;19(1):nsae038. doi: 10.1093/scan/nsae038 (PMC11192622; doi:10.1093/scan/nsae038)
Supplement: nsae038_Supp [file nsae038_supp.zip › scan-24-006-File005.docx]

Supplementary Material

**The scent of cuteness - Neural signatures of infant body odors**

*Laura Schäfer^1^, Carina Köppel^1,2^, Denise Kreßner-Kiel^1^, Sarah Schwerdtfeger^1^, Marie Michael^3^, Kerstin Weidner^1^, Ilona Croy^145^*

^1^ Department of Psychotherapy and Psychosomatic Medicine, Faculty of Medicine, Technische Universität Dresden, Fetscherstraße 74, 01307 Dresden, Germany

^2^ Charité - Universitätsmedizin Berlin, corporate member of Freie Universität Berlin and Humboldt- Universität zu Berlin, Department of Psychiatry and Psychotherapy, Hindenburgdamm 30, 12203 Berlin, Germany

^3^ Max Planck School of Cognition, Stephanstr 1a, Leipzig, Germany

^4^ Department of Clinical Psychology, Institute of Psychology, Friedrich-Schiller-Universität Jena

^5^ German Center for Mental Health (DZPG), Site Jena-Magdeburg-Halle

*Supplementary Methods*

Body odor sampling**:**

Body odors were sampled from onesies or T-shirts (100% made of cotton wool), which the infant/postpubertal donor wore for one night during sleeping. The study kit contained the garment in the respective size of the child (prior washed with odorless detergent: denkmit® Ultra sensitive Vollwaschmittelpulver); as well as odorless detergent to wash sheets and extra clothes of the child (e.g. pyjama) prior to the experimental night, odorless shower el (Eubos ® Flüssig Duschhautpflege) and a plastic zip-bag. We instructed the mothers to wash their infant and the postpubertal children to wash themselves with the shower gel the evening before the experimental night and refrain from using perfumed products. The following morning, the garments were stored in the zip-bag and brought back to the lab, where both armpit areas were cut and frozen at -25°C. Garments were thawed 2 hours prior to the experiment.

Preprocessing:

BOLD extraction analyses: The DICOM files were imported to NifTI-format by the conversion utility implemented in SPM 12 (Wellcome Trust Center for Neuroimaging, London, UK, implemented in Matlab R2017b, MathWorks, Inc., Natick, Ma, USA). For the preprocessing, we used the default settings implemented in SPM 12. The functional and structural images were realigned with a 2nd degree B-spline and unwarped with a 4th degree B-spline. This was followed by co-registration and segmentation fitting to the individual T1 volume. The realigned images were normalized into MNI ICBM 152-space using 4th degree B-Spline interpolation. Thereafter, we smoothed the normalized images using a FHWM Gaussian kernel of 4mm to ensure specificity while increasing sensitivity for our smaller ROIs [1].

Functional connectivity analyses: First, the DICOM files [2] were imported to NII-format by application of the dcm2nii.exe which runs under MRICroGl . Second, the structural and functional data were entered in the CONN Setup GUI, together with the following covariates: pleasantness-/wanting-/intensity-ratings and identification performance. Group was added as a covariate (mothers, nulliparae). Conditions were specified for each participant and each run. The pre-defined ROIs were entered in the Setup step. Third, the preprocessing of the fMRI data was performed according to the standard preprocessing pipeline implemented in the CONN toolbox [3] , including functional realignment and unwarp, slice time correction, outlier identification, segmentation and normalization, functional smoothing (4mm FHWM). Subsequently, the denoising of the functional data was performed. This included the linear regression of five principal components from CSF (cerebro-spinal fluid), five principal components from white matter, 12 principal components from realignment and 127 principal components from scrubbing, and two principal components per condition. An additional step was linear detrending. To minimize the effects of respiratory and cardiac noise, a temporal band-pass filter of 0.01 to 0.1Hz was added to the time series [4, 5].

*
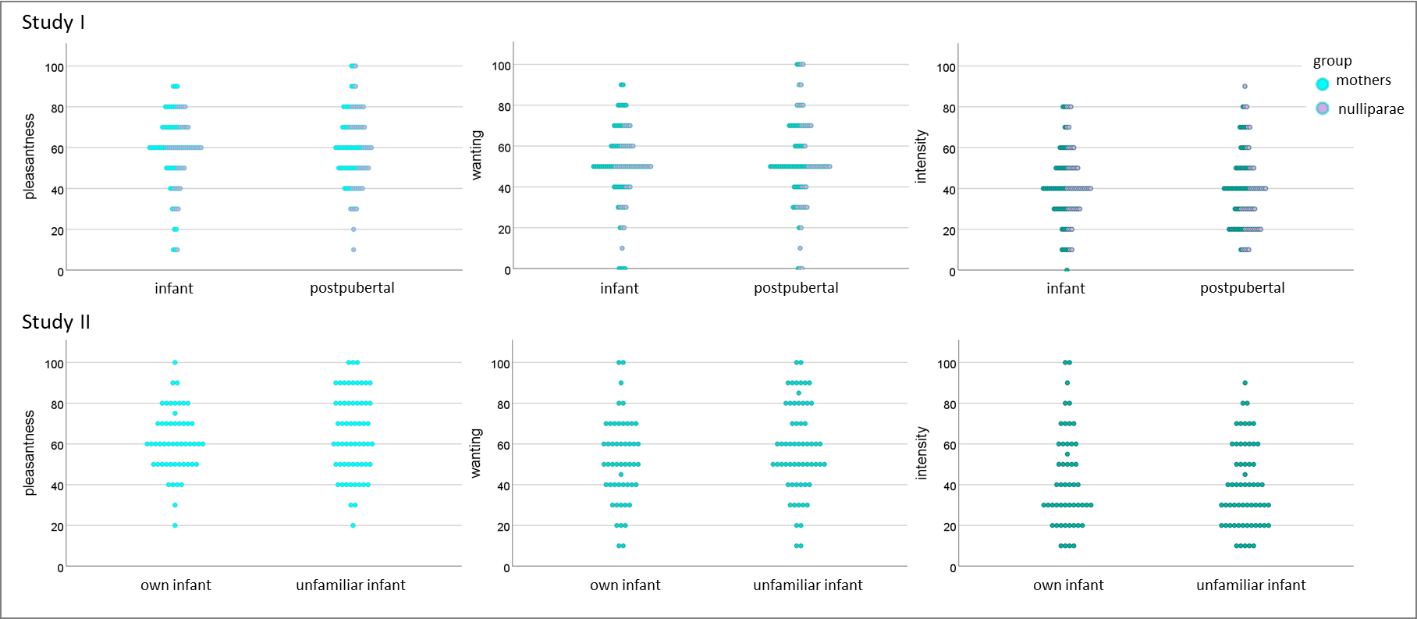
Supplementary Figures*

*Figure S1*. Perceptual ratings (pleasantness, intensity and wanting scores) ranging from 0 – “not at all” to 100 “very” of the presented body odor conditions depicted per group and condition (study 1) and per condition (study 2). Study I: Green dots display perceptual ratings of mothers, lilac dots display perceptual ratings of nulliparae.


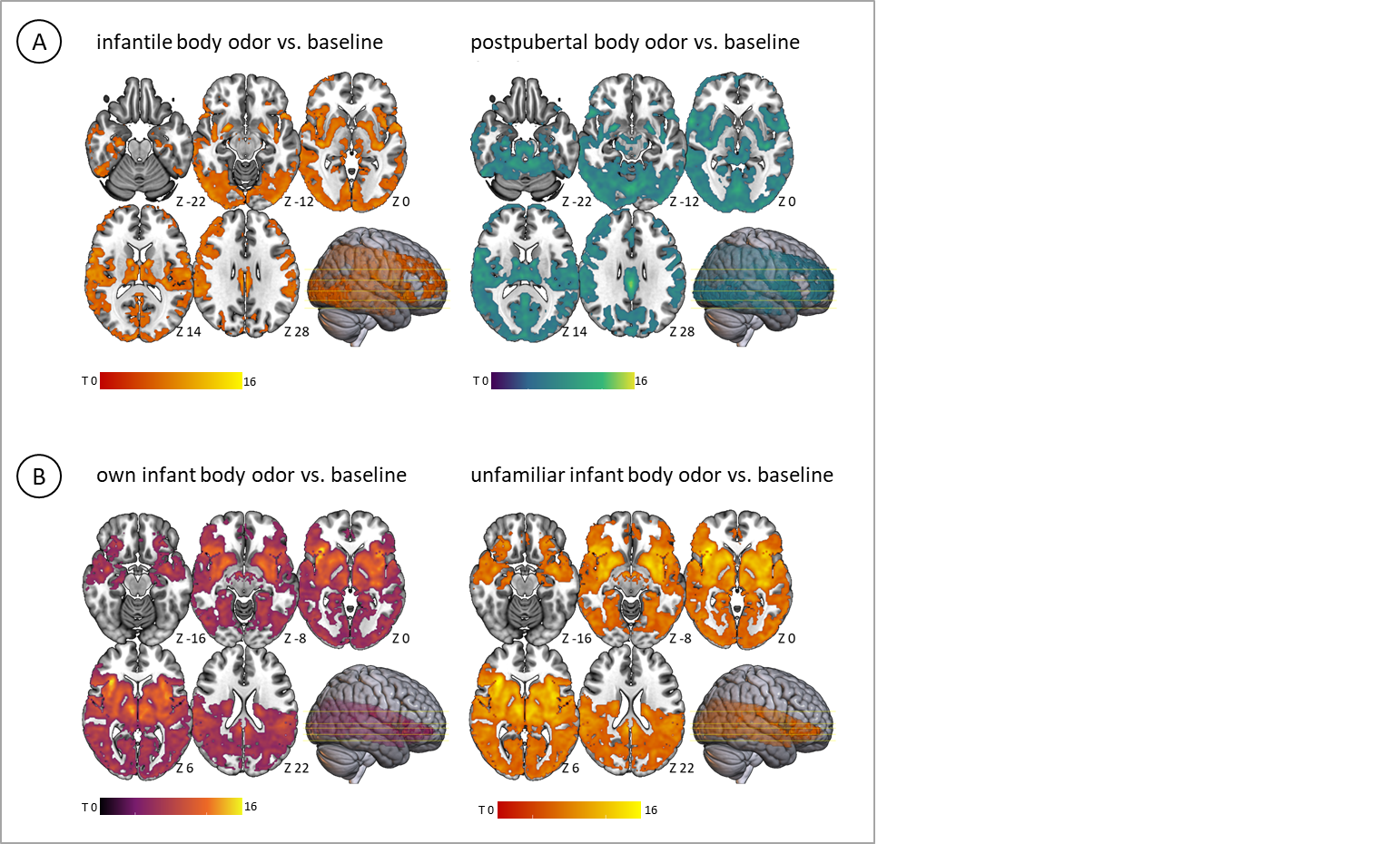


*Figure S2*. Whole Brain analysis of the body odor conditions show a significant BOLD signal increase over multiple brain areas, encompassing the pleasure, reward and olfaction network (height threshold: *p* < 0.05, *FWE* corrected). A) study I: unfamiliar infant body odor vs. baseline; postpubertal body odor vs. baseline; B) study II: own infant body odor vs. baseline; unfamiliar infant body odor vs. baseline. Z- range from -22 to 22, colorbars represent T-values from lowest (T=0) to highest (T=16).

**
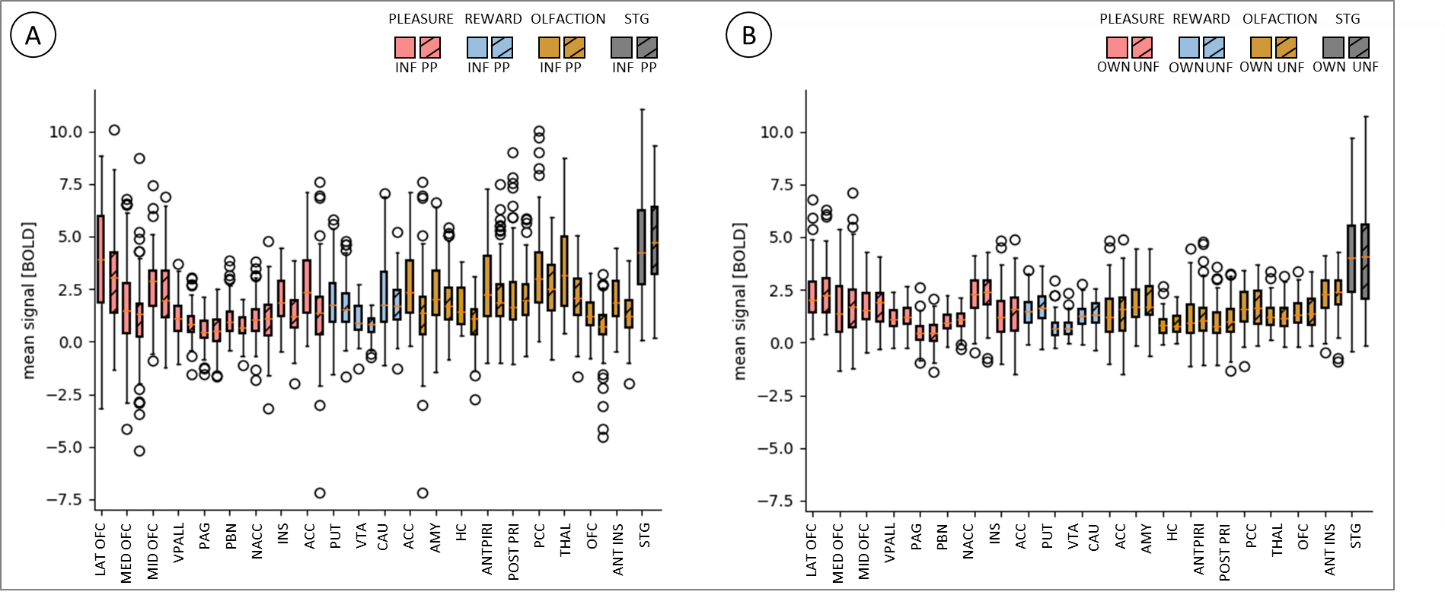
**

Figure S3. A) Mean BOLD signal extracted values plotted per ROI and condition for the respective networks of interest show higher (* = p < 0.05) signal strength after smelling infantile vs postpubertal body odors in each of the networks (compare Fig. 2C). B) Mean BOLD signal extracted values plotted per ROI and condition for the respective networks of interest show no significant difference after smelling body odors of the own vs. an unfamiliar infant (compare Fig. 2D). Error bars depict 95% confidence interval. Abbreviations: INF = infantile body odor, PP = postpubertal body odor, OWN = own infant body odor, UNF = unfamiliar infant body odor; networks and ROIs: pleasure: LAT OFC = lateral orbitofrontal cortex, MED OFC = medial orbitofrontal cortex, MID OFC = mid-anterior orbitofrontal cortex, VPALL = ventral pallidum, PAG = periaqueductal grey, pBN = parabrachial nucleus, NACC = nucleus accumbens, INS = insula, ACC = anterior cingulate cortex; reward: PUT = putamen, CAU = caudate, VTA = ventral tegmentum area; olfaction: ACC = anterior cingulate cortex, AMY = amygdala, HC = hippocampus, PIRI = piriform cortex, PCC = posterior cingulate cortex, THAL = thalamus, OFC = orbitofrontal cortex, ANT INS = anterior insula, social cognition: STG = superior temporal gyrus.

*Supplementary Tables*

Table S1. *Perceptual ratings of body odor stimuli*

|  |  | **group** | **condition** | **pleasantness** | |  | **wanting** | |  | **intensity** | | |
| --- | --- | --- | --- | --- | --- | --- | --- | --- | --- | --- | --- | --- |
|  |  |  |  | ***M*** | ***SD*** |  | ***M*** | ***SD*** |  |  | ***M*** | ***SD*** |
| Study I |  | mothers | infant | 59.75 | 20.32 |  | 51.75 | 23.45 |  |  | 39.00 | 18.29 |
|  |  |  | postpubertal | 61.90 | 15.64 |  | 52.70 | 17.79 |  |  | 41.63 | 16.95 |
|  |  | nulliparae | infant | 57.25 | 15.34 |  | 48.50 | 13.71 |  |  | 43.25 | 17.39 |
|  |  |  | postpubertal | 57.49 | 20.15 |  | 52.56 | 24.24 |  |  | 37.28 | 20.09 |
| Study II |  | mothers | infant own | 61.83 | 15.86 |  | 52.20 | 20.65 |  |  | 42.53 | 23.07 |
|  |  |  | infant unfamiliar | 64.95 | 159.81 |  | 57.81 | 22.00 |  |  | 39.53 | 20.31 |

*Note.* Study I: A generalized linear univariate model was calculated for each perceptual rating (pleasantness, wanting, intensity) to examine differences with regard to group or condition (per study). The rating served as target, model specifications were robust estimations, and the identity function was used as the linking function according to the linear model specifications. The following effects were modeled: study I: main effect of condition (infant vs. postpubertal odor) and main effect of group (mothers vs. nulliparae); study II: main effect of condition (infant vs. postpubertal odor). Perceptual ratings differed neither significantly between condition nor group (pleasantness: *F*(2,2) = .79, *p* =.550; wanting: *F*(2,2) = .1.06, *p* = .346; intensity: *F*(2,2) = .13, *p* = .881). Study II: Perceptual ratings did not differ significantly between condition (pleasantness: *F*(1,6) = 0.48, *p* =.517; wanting: *F*(1,6) = 1.51, *p* = .265; intensity: *F*(1,6) = 0.25, *p* = .632).

Table S2*. Study II: sensitivity, specificity, and accuracy of kin identification*

|  |  |  |  |  | **sensitivity** |  | **specificity** | **accuracy** |
| --- | --- | --- | --- | --- | --- | --- | --- | --- |
|  | *tp* | *tn* | *fp* | *fn* | *tp/(tp+fn)* |  | *tn/(tn+fp)* | *tp+tn/(all)* |
| run 1 | 2 | 13 | 1 | 12 | 0,14285714 |  | 0,92857143 | 0,53571429 |
| run 2 | 7 | 9 | 5 | 11 | 0,38888889 |  | 0,64285714 | 0,5 |
| run 3 | 4 | 18 | 3 | 8 | 0,33333333 |  | 0,85714286 | 0,66666667 |
| run 4 | 3 | 8 | 6 | 11 | 0,21428571 |  | 0,57142857 | 0,39285714 |
| across all runs | 16 | 48 | 15 | 42 | 0,27586207 |  | 0,76190476 | 0,52892562 |

Table S3. *Characteristics of the* *regions of interest (ROI) with their respective assignment to the network and the atlases for mask creation. The last column displays the coordinates of the peak voxel (extracted from MNI space, XYZ coordinates) for study I and II. Those peak voxels served as center for a 4mm sphere extraction (cluster size: 264 voxel per sphere).*

| ROI | **assigned network** | | | **atlas** | **extracted from MNI X Y Z [study I; study II]** |
| --- | --- | --- | --- | --- | --- |
|  | **olfaction** | **pleasure** | **reward** |  |  |
| amygdala  piriform cortex  thalamus hippocampus  posterior cingulate cortex  orbitofrontal cortex  anterior cingulate cortex  insula  lateral orbitofrontal cortex  mid anterior orbitofrontal cortex  medial orbitofrontal cortex  nucleus accumbens  ventral pallidum  periaqueductal grey  parabrachial nucleus  ventral tegmentum area  caudate  putamen  superior temporal gyrus | X  X  X  X  X  X  X  X | X  X  X  X  X  X  X  X  X | X  X  X | AAL  iMRIcron^1^  ibaspm 116  ibaspm 116  ibaspm 116  ibaspm 116  ibaspm 116  ibaspm 116  ibaspm 71  ibaspm 71  ibaspm 71  ibaspm 71  ibaspm 71  AAN  AAN  AAN  ibaspm 71  ibaspm 71  ibaspm 116 | -24 0 -12; -24 -2 -12  -22 0 -14; -22 0 -10  4 -20 10; 8 -16 6  28 -16 -22; -20 -38 -2  0 -34 28; 4 -42 22  -36 29 -13; 2 48 -4  0 30 26; 2 42 -13  40 -18 -2; 34 20 6  48 22 -10; -38 26 -4  48 8 38; -38 58 -6  -6 64 -16; 2 54 -8  14 4 -14; 12 8 -6  -22 2 0; -12 8 -2  -4 -30 -6; -4 -32 -10  -8 -32 -24; --^a^  -2 -24 -20 ; 0 -22 -16  12 -2 14;4 12 12  -24 0 -10; -24 2 -8  56 18 -8; -56 12 -2 |

^1^ as guided by [6] [McCausland Center for Brain Imaging, <https://www.mccauslandcenter.sc.edu/crnl/mricron>], ^a^ no above threshold activations observed

Table S4. *Results of BOLD signal extraction per network*

| **study** | **group** | **body odor condition** | **network** | **condition vs baseline** | **effect of condition** | **effect of group** |
| --- | --- | --- | --- | --- | --- | --- |
| I | all subjects | infantile | olfactory | *t*(39) = 16.01, *p* <.001,  *d* = 2.53 | *F*(1,804) = 45.42, *p* =.001 |  |
|  |  |  | reward | *t*(39) = 10.69, *p* <.001,  *d* = 1.69 | *F*(1,192) = 10.34, p =.002 |  |
|  |  |  | pleasure | *t*(39) = 15.85, *p* <.001,  *d* = 2.51 | *F*(1,762) = 32.59, *p* <.001 |  |
|  |  |  | stg | *t*(39) = 11.58*, p* <.001,  *d* = 1.83 | *F*(1,117) = 0.48, *p* = .827 |  |
|  |  | postpubertal | olfactory | *t*(39) = 15.70, *p* <.001,  *d* = 2.48 |  |  |
|  |  |  | reward | t(39) = 14.47, p <.001,  d = 2.11 |  |  |
|  |  |  | pleasure | *t*(39) = 10.01, *p* <.001,  *d* = 1.58 |  |  |
|  |  |  | stg | *t*(39) = 15.09, *p* <.001,  *d* = 2.39 |  |  |
|  | mothers | infantile | olfactory | *t*(19) = 10.74, *p* <.001,  *d* = 2.40 |  | *F*(1,37) = 0.60, *p* =.445 |
|  |  |  | reward | *t*(19) = 7.58, *p* <.001,  *d* = 1.70 |  | *F*(1,37) =.05, *p* =.821 |
|  |  |  | pleasure | *t*(19) = 11.27, *p* <.001,  *d* = 2.52 |  | *F(*1,37) =.07, *p* =.878 |
|  |  |  | stg | *t*(19) = 8.82, *p* <.001,  *d* = 1.97 |  | *F*(1, 37) = .11, *p* =.741 |
| … |  | postpubertal | olfactory | *t*(19) = 8.85, *p* <.001,  *d* = 1.97 |  |  |
|  |  |  | reward | *t*(19) = 8.85, *p* <.001,  *d* = 1.98 |  |  |
|  |  |  | pleasure | *t*(19) = 6.47, *p* <.001,  *d* = 1.44 |  |  |
|  |  |  | stg | *t*(19) = 11.25, *p* <.001,  *d* = 2.5 |  |  |
|  | nulliparae | infantile | olfactory | *t*(19) = 11.74, *p* <.001,  *d* = 2.63 |  |  |
|  |  |  | reward | *t*(19) = 7.34, *p* <.001,  *d* = 1.62 |  |  |
|  |  |  | pleasure | *t*(19) = 10.98, *p* <.001,  *d* = 2.45 |  |  |
|  |  |  | stg | *t*(19) = 7.57, *p* <.001,  *d* = 1.69 |  |  |
|  |  | postpubertal | olfactory | *t*(19) = 10.45, *p* <.001,  *d* = 2.33 |  |  |
|  |  |  | reward | *t*(19) = 9.89, *p* <.001,  *d* = 2.21 |  |  |
|  |  |  | pleasure | *t*(19) = 7.67, *p* <.001,  *d* = 1.72 |  |  |
|  |  |  | stg | *t*(19) = 9.98, *p* <.001,  *d* = 2.23 |  |  |
| II | mothers | own infant | olfactory | *t*(37) = 13.40, *p* <.001,  *d* = 2.17 | *F*(1,404) = 3.55, *p* =.060 | - |
|  |  |  | reward | *t*(37) = 15.96, *p* <.001,  *d* = 2.59, *d* = 1.79 | *F*(1,362) = .70, *p* =.417 | - |
|  |  |  | pleasure | (37) = 13.90, *p* <.001,  *d* = 2.15 | *F*(1,595) =3.60, *p* =.058 | - |
|  |  |  | stg | *t*(37) = 11.43, *p* <.001,  *d* = 1.85 | *F*(1,149) =.00, *p* =1.000 | - |
|  |  | unfamiliar infant | olfactory | *t*(37) = 13.36, *p* <.001,  *d* = 2.17; |  | - |
|  |  |  | reward | *t*(37) = 14.48, *p* <.001,  *d* = 2.35 |  | - |
|  |  |  | pleasure | *t*(37) = 13.70, *p* <.001,  *d* = 2.22 |  | - |
|  |  |  | stg | *t*(37) = 10.57, *p* <.001,  *d* = 1.72 |  | - |

*Note.* Abbreviations: stg = superior temporal gyrus

Table S5*. Pearson Correlation coefficients for bivariate relationships between extracted BOLD signal and perceptual ratings and per study, network and condition; and F, df and p-values for the main effect of correct identification of the own infant´s body odor in study II.*

| **study** | **network** | **group** | **body odor condition** | **pleasantness** | | **wanting** | | **correct recognition of own child** | | |
| --- | --- | --- | --- | --- | --- | --- | --- | --- | --- | --- |
|  |  |  |  | *r* | *p* | *r* | *p* | *F* | *df* | *p* |
| I | olfaction | across both | across both | .00 | .969 | -.01 | .468 |  |  |  |
|  | pleasure |  |  | .04 | .109 | .06* | .029 |  |  |  |
|  | reward |  |  | .01 | .863 | .01 | .755 |  |  |  |
|  | stg |  |  | .03 | .768 | .07 | .471 |  |  |  |
|  | olfaction |  |  | -..03 | .328 | -.00 | .986 | 1.05 | 2;1004 | .359 |
|  | pleasure |  |  | -.02 | .497 | -.01 | .597 | 3.07 | 2;502 | .048 |
|  | reward |  |  | .06 | .274 | .04 | .517 | 1.18 | 2;281 | .309 |
|  | stg |  |  | -.^^ | .314 | -.^0 | .370 | 0.35 | 2;148 | .702 |

*Note.* Abbreviations: stg = superior temporal gyrus

Table S6. *Results of ROI-to-ROI functional connectivity analysis: Between-group and between-conditions contrasts and results of covariate analyses (perceptual ratings)*

| **study** | **network** | **group** | **condition** | **covariate** | **seed-ROI** | **target-ROI** | ***T*** | ***p_FDR_*** |
| --- | --- | --- | --- | --- | --- | --- | --- | --- |
| study I | pleasure | mothers > nulliparae | infant |  | med ofc | pbn | 3.66 | .009 |
|  | stg - pleasure | all subjects | infant > postpubertal |  | stg | pag | 3.63 | .010 |
| study II | reward - olfaction | all subjects | own infant |  | caudate | pcc | 3.52 | .001 |
|  | pleasure | all subjects | own infant |  | vpall | mid ofc | 3.11 | .004 |
|  |  |  |  |  | vpall | insula | 2.89 | .006 |
|  |  |  |  |  | vpall | nacc | 2.04 | .048 |
|  |  |  |  |  |  |  |  |  |
| study I | pleasure | all subjects | average body odor | pleasantness | vpall | med ofc | 2.74 | .045 |
|  |  |  |  |  | vpall | lat ofc | 2.67 | .046 |
| study II | pleasure | all subjects | average body odor | pleasantness | lat ofc | mid ofc | 3.25 | .021 |

*Note*. Abbreviations: stg = superior temporal gyrus, med ofc = medial orbitofrontal cortex, pbn = parabrachial nucleus, pag = periaqueductal grey, pcc = posterior cingulate cortex, vpall = ventral pallidum, mid ofc = mid anterior orbitofrontal cortex, nacc = nucleus accumbens, lat ofc = lateral orbitofrontal cortex

Table S7. *Sample characteristics*

|  | **study I** | | | **study II** | | |
| --- | --- | --- | --- | --- | --- | --- |
|  | *M* | *SD* | *n* | *M* | *SD* | *n* |
| age mothers (years) | 32.2 | 3.8 | 20 | 32.1 | 3.1 | 38 |
| age nulliparae (years) | 24.2 | 1.2 | 20 | -- | -- | 38 |
| age infant body odor donors (months), 14 girls, 11 boys (stI); 18 girls, 15 boys (stII) | 22.1 | 11.5 | 14^a^;11 ^b^ | 22.9 | 9.0 | 18^a^;15^b^ |
| age postpubertal body odor donors (years), 6 girls, 7 boys | 15 | 1.5 | 6^a^;7^b^ | -- | -- | -- |
| number of biological children | 1.65 | 0.7 | 20 | 1.59 | 0.6 | 38 |
| smoking(years) in the past | 8.08 | 4.4 | 6 | 8.41 | 2.1 | 12 |
| bdi II | 4.54 | 4.1 | 37 | 5.64 | 4.8 | 34 |
| breastfeeding in month | 16.73 | 10.4 | 16 | 12.17 | 7.12 | 30 |
| relationship quality | 4.95 | 0.2 | 20 | 4.71 | 0.9 | 34 |
| relationship closeness | 4.90 | 0.3 | 20 | 4.89 | 0.5 | 34 |
| distribution of parental care | 62.25 | 18.8 | 20 | 68.14 | 15.2 | 34 |
| SDI score | 13.75^*^ | 1.4 | 40 | 3^**^ | 0.0 | 38 |

*Note.* ^a^female; ^b^male;  ^*^total of 16 sniffin sticks; ^**^ total of 3 identification scores [7]

Table S8. *Results of Whole Brain Analyses and small volume corrections, cluster extent threshold k > 3, p_FWE_ <.05, peak height threshold, areas assigned with SPM Anatomy toolbox [8]*

| **study** | **condition** | **side** | **area** | **cluster** | **coordinates** | | |  |  |  |  |
| --- | --- | --- | --- | --- | --- | --- | --- | --- | --- | --- | --- |
|  |  |  |  | ***k*** | ***X*** | ***Y*** | ***Z*** | ***T*** | ***p_FWE_*** | ***p_FDR_-*** | ***p_unc_*** |
| I | infantile body odor | right | temporal pole | 55582^*^ | 56 | 18 | -6 | 15.37 | 0 | 2.1697E-10 | 4.4409E-16 |
|  |  | left | gyrus precentralis | 27 | -20 | -26 | 56 | 7.99 | 3.0417E-08 | 4.4075E-06 | 1.5921E-13 |
|  |  | right | primary somatosensory cortex | 39 | 28 | -34 | 54 | 7.62 | 2.4427E-07 | 2.6851E-05 | 1.277E-12 |
|  |  | right | area Fo3 | 61 | 24 | 32 | -18 | 7.39 | 8.6575E-07 | 7.8871E-05 | 4.5254E-12 |
|  |  | right | motorcortex | 22 | 22 | -26 | 56 | 7.12 | 3.8072E-06 | 0.00028266 | 1.99E-11 |
|  |  | left |  |  | -46 | 54 | -12 | 5.90 | 0.00211588 | 0.06635152 | 1.1059E-08 |
|  |  | left |  | 2 | -54 | 2 | -34 | 6.20 | 0.00049974 | 0.01942553 | 2.6121E-09 |
|  |  | left | gyrus frontalis inferior | 14 | -48 | 46 | -4 | 6.19 | 0.00050355 | 0.01953305 | 2.632E-09 |
|  |  | left | gyrus postcentralis | 13 | -34 | -34 | 54 | 6.10 | 0.00081425 | 0.02945908 | 4.256E-09 |
|  |  | left | gyrus occipitalis medius | 10 | -38 | -74 | 16 | 5.99 | 0.00140929 | 0.04696911 | 7.3662E-09 |
|  |  | left | gyrus occipitalis superior | 12 | 22 | -94 | 28 | 5.90 | 0.00212851 | 0.06662318 | 1.1125E-08 |
|  |  | left | gyrus frontalis superior | 7 | -24 | -8 | 52 | 5.72 | 0.00520333 | 0.14309474 | 2.7197E-08 |
|  |  | left | lobus parietalis superior | 14 | -24 | -66 | 40 | 5.70 | 0.00577981 | 0.15661462 | 3.021E-08 |
|  |  | right | hippocampus | 3 | 36 | -26 | -16 | 5.69 | 0.00612654 | 0.16438424 | 3.2023E-08 |
|  | postpubertal body odor | right | temporal pole | 74369^**^ | 56 | 18 | -6 | 15.43 | 0 | 1.8321E-10 | 4.4409E-16 |
|  |  | left | gyrus precentralis | 38 | -20 | -28 | 56 | 8.68 | 5.3102E-10 | 1.3135E-07 | 2.9976E-15 |
|  |  | right | gyrus orbitalis superior | 124 | 24 | 30 | -18 | 7.41 | 7.6808E-07 | 6.5416E-05 | 4.0149E-12 |
|  |  |  |  |  | 36 | 38 | -16 | 6.56 | 7.409E-05 | 0.00335329 | 3.8726E-10 |
|  |  | left | inferior parietal cortex | 5 | -44 | -82 | 20 | 6.24 | 0.00039252 | 0.01434243 | 2.0517E-09 |
|  |  | left | gyrus frontalis inferior | 11 | -24 | 10 | -24 | 6.18 | 0.00051546 | 0.01815607 | 2.6942E-09 |
|  |  | left | hippocampus | 5 | -26 | -40 | -4 | 5.87 | 0.00242818 | 0.07102205 | 1.2692E-08 |
|  |  | left | gyrus frontalis medius | 9 | -28 | 50 | 20 | 5.73 | 0.00495275 | 0.1325044 | 2.5887E-08 |
|  |  | right | hippocampus | 5 | 36 | -26 | -16 | 5.69 | 0.00583445 | 0.15231583 | 3.0496E-08 |
|  |  | left | gyrus precentralis | 9 | -26 | -6 | 48 | 5.53 | 0.01256534 | 0.29692077 | 6.5677E-08 |
|  |  | left | posterior cingulate cortex | 6 | -10 | -50 | 30 | 5.42 | 0.021383 | 0.47105801 | 1.1177E-07 |
| II | own infant bdy odor | left | insula | 61044^***^ | 34 | 22 | 6 | 16.73 | 0 | 5.24824E-11 | 4.44E-16 |
|  |  | left | anterior cingulate gyrus | 452 | 0 | 42 | -2 | 7.30 | 8.2432E-07 | 5.8151E-05 | 8.1012E-12 |
|  |  | left | frontal pole | 34 | 24 | 58 | -8 | 6.54 | 4.6893E-05 | 0.00210865 | 4.6084E-10 |
|  |  | left | frontal pole | 7 | 28 | 54 | -12 | 5.69 | 0.00334028 | 0.09238158 | 3.2826E-08 |
|  | unfamiliar infant body odor | left | insula | 63334^****^ | 34 | 20 | 6 | 17.50 | 0 | 5.1096E-11 |  |
|  |  | left | anterior cingulate gyrus | 724 | 0 | 42 | -2 | 8.28 | 3.4908E-09 | 4.455E-07 |  |
|  |  | right | frontal pole | 4 | -22 | 60 | -10 | 5.99 | 0.00077752 | 0.02565994 |  |
|  |  | right | inferior temporal gyrus | 3 | -48 | -56 | -12 | 5.73 | 0.00277256 | 0.07874432 |  |

*Note.* Whole-brain analyses were conducted (*p_FWE_* <.05) for the main contrasts (study I: infantile body odor vs baseline, postpubertal body odor vs. baseline; study II: own infant body odor vs. baseline, unfamiliar infant body odor vs. baseline (see Figure S2). For the whole brain analyses, minimal voxel cluster size was restricted to *k* = 3 [9]. *Activation maxima primarily originating from the temporal lobe, but also associated with the cingulate cortex, thalamus, and cerebellum. ** Activation maxima primarily localized in the temporal lobe, the superior temporal gyrus, somatosensory cortex and primary motor cortex. ***Activation maxima in the thalamus, frontal operculum cortex, precentral gyrus. ****Activation maxima in the thalamus, frontal operculum cortex, precentral gyrus, putamen, pallidum.

The additional supplementary datasets SD1-8 are uploaded on <https://figshare.com/s/9a937a9fa3be8df6e480>.

**Supplementary references**

1. Morawetz, C., et al., *Improved functional mapping of the human amygdala using a standard functional magnetic resonance imaging sequence with simple modifications.* Magnetic resonance imaging, 2008. **26**(1): p. 45-53.

2. Rorden, C. and M. Brett, *Stereotaxic display of brain lesions.* Behavioural neurology, 2000. **12**(4): p. 191-200.

3. Whitfield-Gabrieli, S. and A. Nieto-Castanon, *Conn: a functional connectivity toolbox for correlated and anticorrelated brain networks.* Brain connectivity, 2012. **2**(3): p. 125-141.

4. Cordes, D., et al., *Characterization and reduction of cardiac-and respiratory-induced noise as a function of the sampling rate (TR) in fMRI.* Neuroimage, 2014. **89**: p. 314-330.

5. Wee, C.-Y., et al., *Resting-state multi-spectrum functional connectivity networks for identification of MCI patients.* PloS one, 2012. **7**(5): p. e37828.

6. Zelano, C., et al., *Attentional modulation in human primary olfactory cortex.* Nature neuroscience, 2005. **8**(1): p. 114.

7. Lötsch, J., A. Ultsch, and T. Hummel, *How Many and Which Odor Identification Items Are Needed to Establish Normal Olfactory Function?* Chemical senses, 2016. **41**(4): p. 339-344.

8. Eickhoff, S.B., et al., *A new SPM toolbox for combining probabilistic cytoarchitectonic maps and functional imaging data.* Neuroimage, 2005. **25**(4): p. 1325-1335.

9. Lundström, J.N., et al., *Maternal status regulates cortical responses to the body odor of newborns.* Frontiers in psychology, 2013. **4**.
